# Supplementary figures and images for: Proteomic analysis of rat serum revealed the effects of chronic sleep deprivation on metabolic, cardiovascular and nervous system
Source: PLoS One. 2018 Sep 20;13(9):e0199237. doi: 10.1371/journal.pone.0199237 (PMC6147403; doi:10.1371/journal.pone.0199237)

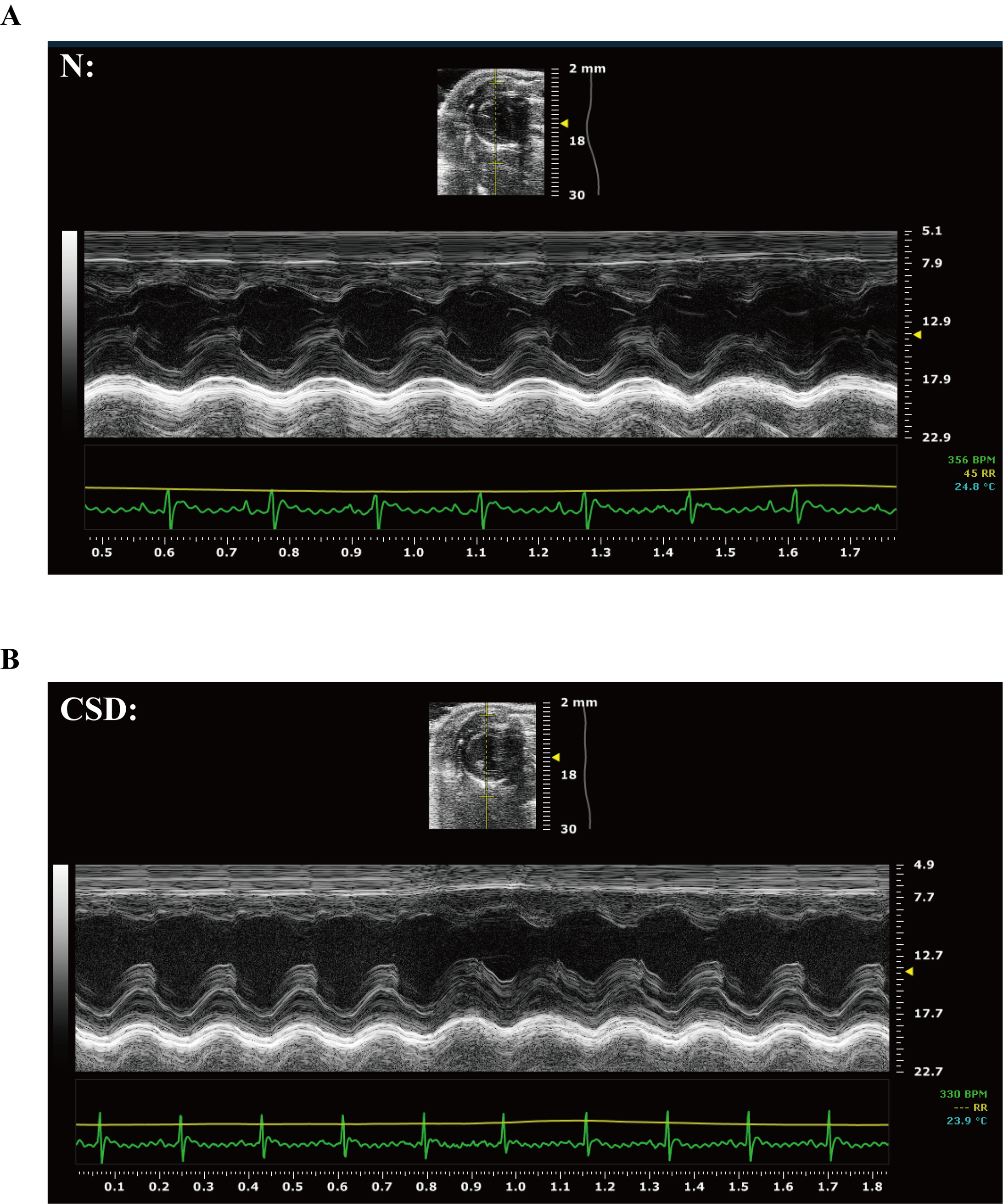

Supplement: S1 Fig — Posterior wall of the left ventricle was significantly thickening in the CSD-group rat. (TIF) [file pone.0199237.s001.tif]
