# Supplementary material for: Proteomic analysis of rat serum revealed the effects of chronic sleep deprivation on metabolic, cardiovascular and nervous system
Source: PLoS One. 2018 Sep 20;13(9):e0199237. doi: 10.1371/journal.pone.0199237 (PMC6147403; doi:10.1371/journal.pone.0199237)
Supplement: S2 Table — (DOCX) [file pone.0199237.s003.docx]

Table S2. The four candidates verified in PRM study

| Protein name | Gene | Uniprot ID | Peptide sequence |
| --- | --- | --- | --- |
| Pyruvate kinase PKM | Pkm | P11980 | VNLAMNVGK  EAEAAVFHR |
| Clusterin | Clu | Q6P7S6 | EIQNAVQGVK  SLLNSLEEAK |
| Kininogen-1 | Kng1 | P08934 | FSIATQICNITPGK  NIPVDSPELK  ATSQVVAGTK |
| Profilin-1 | Pfn1 | P62963 | DSPSVWAAVPGK  TFVSITPAEVGVLVGK |
